# Study on Dynamic Mechanical Behavior of 34CrNi3MoA Alloy Steel Considering the Coupling Effect of Temperature and Strain Rate

**DOI:** 10.3390/ma18204658

**Published:** 2025-10-10

**Authors:** Xiaoyan Guan, Zhengyuan Zhang, Hengheng Wu, Jianzhi Chen, Li Sun, Guochao Li

**Affiliations:** School of Mechanical Engineering, Jiangsu University of Science and Technology, Zhenjiang 212003, China

**Keywords:** constitutive model, 34CrNi3MoA, SHPB, temperature softening, strain-hardening

## Abstract

Temperature and strain rate play a crucial role in determining the mechanical properties of metals. These critical parameters are typically assessed using the split Hopkinson pressure bar (SHPB) test. However, previous studies have seldom considered the coupled influence of temperature and strain rate on dynamic mechanical behavior, thereby reducing the accuracy of constitutive models. To accurately characterize the dynamic mechanical behavior of 34CrNi3MoA low-alloy steel, a new constitutive model combining temperature and strain rate was developed. Firstly, SHPB experiments under varying temperatures and strain rates were designed to obtain actual stress–strain curves. The results indicate that the mechanical properties of 34CrNi3MoA low-alloy steel are significantly influenced by both temperature and strain rate. True stress has a significant temperature-softening effect within the temperature range of 25 °C to 600 °C, while the flow stress in the yield stage increases with rising strain rate. Secondly, a novel constitutive model was established by integrating a correction function. The model comprises three components: a strain rate-strengthening function influenced by temperature, a temperature-softening function influenced by strain rate, and a strain-hardening correction function accounting for the coupling of temperature and strain rate. Comparing the mean relative error, the new model significantly improves accuracy compared to the original Johnson–Cook (J-C) model.

## 1. Introduction

34CrNi3MoA is a medium-carbon, low-alloy steel that can undergo quenching and tempering heat treatment. It can be utilized for the impeller disk and impeller cover of air compressors, as well as for gears and engine rotors. It is predominantly employed in large steam turbines, air compressors, and other equipment. The dynamic mechanical properties of 34CrNi3MoA have a great influence on the machinability and service life of 34CrNi3MoA low-alloy steel parts. Under varying external conditions such as temperature and strain rate, 34CrNi3MoA exhibits distinct dynamic mechanical properties. Consequently, a systematic and comprehensive investigation of the dynamic mechanical properties of 34CrNi3MoA low-alloy steel at diverse temperatures and strain rates is crucial for optimizing and enhancing the machining process of this material.

As an engineering material with extensive applications, 34CrNi3MoA low-alloy steel has been the subject of numerous studies on its constitutive relationships under various deformation conditions. The study by Bao et al. on 38CrMoAl steel shows that its yield strength increases with the strain rate (850~4500 s^−1^) [1], but the temperature softening effect at high strain rates can offset part of the strengthening, resulting in a gradual decrease in the strength growth rate as the strain rate increases. Jia et al. investigated the thermal deformation behavior of 34CrNi3Mo low-alloy steel at high temperatures and low strain rates using a Gleebel-3500 thermal simulation testing machine [2]. Results indicate that its flow stress is jointly influenced by temperature, strain rate, and strain, resulting from the interaction of work hardening, dynamic recovery, and recrystallization. A modified Arrhenius-type constitutive model was established that simultaneously accounts for the coupled effects of strain, temperature, and strain rate, significantly improving the prediction accuracy of flow stress. Niu Qiulin et al. studied the dynamic mechanical behavior of TC17 titanium alloy in the temperature range of 30 °C to 700 °C and strain rates of 3000 s^−1^ to 10,000 s^−1^ through Hopkinson pressure bar experiments [3]. The results showed that the strain rate sensitivity of the alloy increased and then decreased with rising temperature. The temperature sensitivity factor significantly increased below 500 °C, but weakened above 700 °C when the strain rate reached 10,000 s^−1^. Zhang et al. proposed a layer-by-layer inversion method for carburized 18CrNiMo7-6 steel [4], determining depth-dependent Johnson–Cook dynamic constitutive parameters through layered experiments and numerical optimization. The model was validated by finite element simulations and high-temperature dynamic experiments, demonstrating accurate prediction of mechanical responses under high strain rates and elevated temperatures. Zhou et al. systematically investigated the influence of specimen geometric dimensions on the dynamic mechanical behavior of 40Cr alloy steel [5]. They found that true stress decreased significantly with increasing dimension coefficient. The study innovatively established a segmented constitutive model accounting for geometric effects, achieving significantly improved accuracy compared to the traditional J-C model, with a 10.59% reduction in the mean absolute relative error. Jiang et al. investigated the mechanical behavior of AISI 9310 steel under varying hardness, strain rates, and temperatures, revealing pronounced strain rate hardening and temperature softening effects [6]. Consequently, an improved constitutive equation accounting for hardness effects was proposed and validated through shot peening experiments. Yipu Bian et al. proposed an improved JC predictive model suitable for both high and low temperatures for tungsten alloys [7] which overcomes the prediction capability errors of the original JC model at extreme temperatures and promotes the development of finite element simulations for tungsten alloy applications.

Due to strain hardening, the proliferation and entanglement of dislocations during plastic deformation impede further dislocation motion, causing the flow stress of materials to increase with rising strain. Moreover, under high-temperature and high-strain-rate conditions, adiabatic heating generated during deformation activates two key softening mechanisms: dynamic recovery and dynamic recrystallization. Dynamic recovery reduces internal energy through dislocation rearrangement and annihilation, while dynamic recrystallization completely resets the hardened microstructure by forming new, unstrained grains. Both processes influence the dynamic mechanical properties of 34CrNi3MoA low-alloy steel under high-temperature, high-strain-rate conditions. In summary, the classical J-C model isolates strain hardening, strain rate strengthening, and temperature softening, failing to adequately consider the coupling effects among them. This simplification limits the model’s predictive accuracy under a wide range of temperature and strain rate conditions, which has become a major limitation of the model in practical applications.

To more accurately describe the dynamic mechanical properties of 34CrNi3MoA low-alloy steel under high-temperature and high-strain conditions, this article, based on separated Hopkinson bar experiments, investigates for the first time the dynamic mechanical behavior of 34CrNi3MoA low-alloy steel under different strain rates and temperatures. The experiments are primarily divided into two parts: dynamic compression experiments with varying strain rates under the same temperature condition, and dynamic compression experiments with varying temperatures under the same strain rate condition. Based on the true stress–strain curves obtained from dynamic compression experiments and the existing Johnson–Cook (J-C) constitutive model, corresponding correction functions are established by considering thermal softening effects, strain rate strengthening effects, and adiabatic temperature rise. These modifications aim to enable the J-C model to more accurately describe the dynamic mechanical behavior of 34CrNi3MoA low-alloy steel. Although there have been studies proposing modified forms of the J-C model for other types of steel, there is still a lack of dynamic constitutive models specifically for 34CrNi3MoA low-alloy steel. Therefore, this study demonstrates significant novelty in both the material concerned and the model’s adaptability. Finally, the high accuracy and reliability of the modified model are verified through relative error analysis.

## 2. SHPB Experiment

### 2.1. Experimental Design

**Figure 1:**
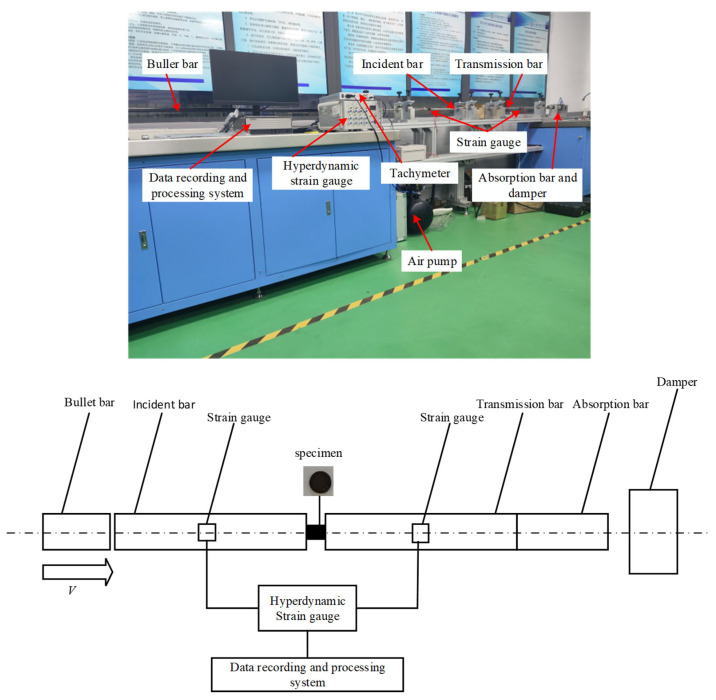
Experimental principle of the SHPB device.

High-strain-rate dynamic compression experiments were conducted using an ALT1000 Split Hopkinson Pressure Bar (SHPB) device made in Archimedes Industry Technology Co., LIMITED, Hong Kong, China, with the experimental principle illustrated in Figure 1. The bullet impacts the incident rod under air gun pressure, generating a compression pulse. Part of this pulse is transmitted to the specimen, inducing high-speed plastic deformation, while another part passes through the specimen into the transmission rod. Eventually, a portion of the pulse is absorbed by the buffer device, while another portion is reflected back to the incident rod.

The experimental material is tempered 34CrNi3MoA low-alloy steel, and its chemical composition is shown in Table 1 [8].

The sample size has a diameter of 5 mm and a length of 3 mm, in the shape of a cylinder. SHPB compression experiments were conducted at four temperatures (25 °C, 200 °C, 400 °C, 600 °C) and four strain rates (1000 s^−1^, 2200 s^−1^, 3500 s^−1^, 4600 s^−1^). The experimental program followed a one-way design with three repetitions per group, resulting in a total of 48 sets of experiments, as shown in Table 2.

### 2.2. SHPB Experimental Principle

The incident strain $\varepsilon_{l}\left(t\right)$, reflected strain $\varepsilon_{R}\left(t\right)$, and transmitted strain $\varepsilon_{r}\left(t\right)$ are measured by strain gauges affixed to the incident and transmission rods, and the stress–strain relationship is deduced. The experimental data were processed using the classical two-wave method to derive the engineering strain ε and engineering stress σ. To eliminate the effect of transverse deformation of the specimen, the engineering stress and engineering strain were converted into true stress $\sigma_{T}$ and true strain $\varepsilon_{T}$ [9,10]. See Equations (1)–(3) for specific calculations.

(1) $$\sigma \left(t\right)=\frac{E_{B}\varepsilon_{T}A_{b}}{A_{S}}$$

(2) $$\varepsilon \left(t\right)=C_{b}/L_{s}\int_{0}^{t}\left(\varepsilon_{l}\left(t\right)-\varepsilon_{R}\left(t\right)-\varepsilon_{T}(t)\right)dt=-2C_{b}/L_{s}\int_{0}^{t}\varepsilon_{R}\left(t\right)dt$$

(3) $$\dot{\varepsilon }\left(t\right)=\frac{C_{b}}{L_{s}\left(\varepsilon_{l}\left(t\right)-\varepsilon_{R}\left(t\right)-\varepsilon_{T}\left(t\right)\right)}=-\frac{2C_{b}}{L_{s}\varepsilon_{R}\left(t\right)}$$

## 3. Experimental Results and Analysis

### 3.1. Temperature Effect Analysis

#### 3.1.1. Analysis of Stress and Strain Curves at Different Temperatures Under the Same Strain Rate

**Figure 2:**
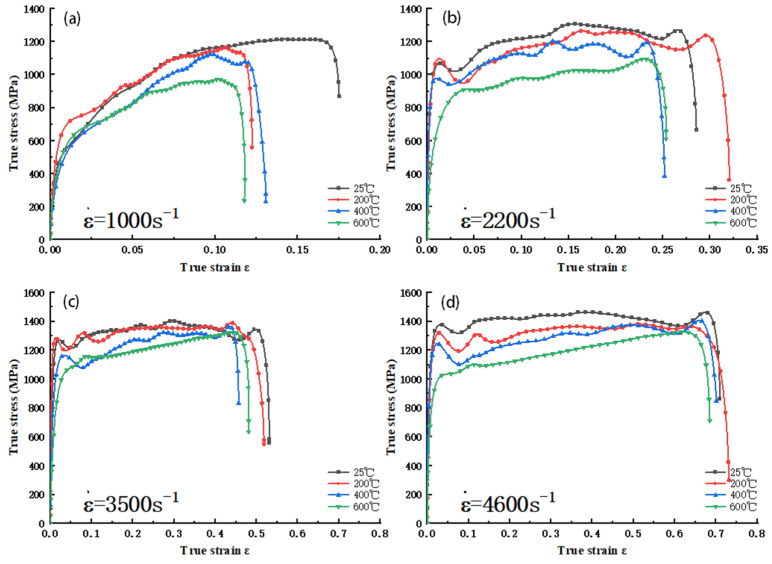
σ−ε curve of 34CrNi3MoA low-alloy steel at different temperatures at the same strain rate: (**a**) ε = 1000 s^–1^; (**b**) ε = 2200 s^–1^; (**c**) ε = 3500 s^–1^; (**d**) ε = 4600 s^–1^.

As illustrated in Figure 2, the dynamic compression impact σ−ε curves of 34CrNi3MoA low-alloy steel at various temperatures and strain rates of 1000 s^−1^, 2200 s^−1^, 3500 s^−1^, and 4600 s^−1^ are presented. It can be observed that the dynamic compression behavior of the 34CrNi3MoA low-alloy steel is qualitatively consistent with that observed in existing studies on similar high-strength steels, exhibiting significant strain hardening and temperature softening phenomena [11,12,13]. Furthermore, the experimental study reveals two new phenomena: first, when the strain rate is 1000 s^−1^, the true stress of the low-alloy steel increases significantly with increasing true strain in the early stage of the plastic phase. However, in the latter half of the plastic phase, the increasing trend of true stress with the rise in true strain slows down or even gradually decreases (Figure 2a). This is largely due to the significant increase in adiabatic temperature caused by plastic strain as the strain increases, making the thermal softening effect more pronounced. When the thermal softening effect exceeds the strain hardening effect, the curve will show a certain downward trend. Second, at strain rates of 2200 s^−1^, 3500 s^−1^, and 4600 s^−1^, the increasing trend of the σ-ε curve during plastic deformation is relatively small, and the strain hardening effect is not significant compared to that at 1000 s^−1^. Meanwhile, with the increase in temperature, the true stress at each strain rate significantly decreases, showing a clear temperature-lowering effect. When the strain rates are 3500 s^−1^ and 4600 s^−1^, the distinction of true stress during the low strain stage is easy, but as the strain increases, in the final stage of strain, there is a notable convergence of the true stress curves at different temperatures (Figure 2c,d), indicating that the temperature-lowering effect weakens with increased strain at high strain rates.

#### 3.1.2. Effect of Temperature on the Compressive Strength of the Material

**Figure 3:**
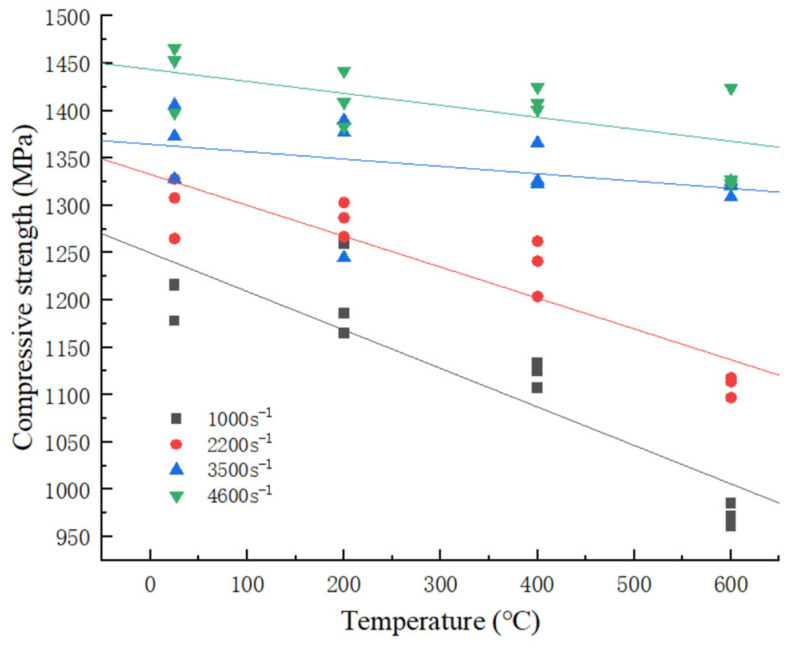
Effect pattern of temperature on compressive strength.

The variation in compressive strength of the 34CrNi3MoA low-alloy steel with temperature is illustrated in Figure 3. As the temperature increases, the compressive strength of the material exhibits a conspicuous decreasing trend. The temperature-induced softening effect is markedly evident in the compressive strength index of the material. As the strain rate increases, it is observed that the rate of decrease in the compressive strength of the material is reduced. This phenomenon is particularly pronounced at strain rates ranging from 2200 s^−1^ to 3500 s^−1^. At elevated strain rates, the dislocation motion of the material becomes more intense, allowing the material to complete the crystal transformation process in a significantly shorter time [14,15]. Consequently, the strength of the material approaches saturation and the influence of temperature on material strength diminishes.

#### 3.1.3. Temperature Sensitivity Analysis

To study the effect of temperature on the mechanical properties of 34CrNi3MoA low-alloy steel, the classical temperature sensitivity factor $S_{T}$ is used, as defined by Equation (4), which has been widely applied in the high strain rate constitutive research of various metal materials.

(4) $$S_{T}=-\frac{\ln\left(\frac{\sigma_{1}}{\sigma_{0}}\right)}{\ln\left(\frac{T_{1}}{T_{0}}\right)}$$

where $T_{0}$ is the reference temperature, taking the room temperature as 25 °C; $T_{1}$ is the experimental ambient temperature; $\sigma_{0}$ and $\sigma_{1}$ are the corresponding flow stresses at $T_{0}$ and $T_{1}$, respectively [16,17].

**Figure 4:**
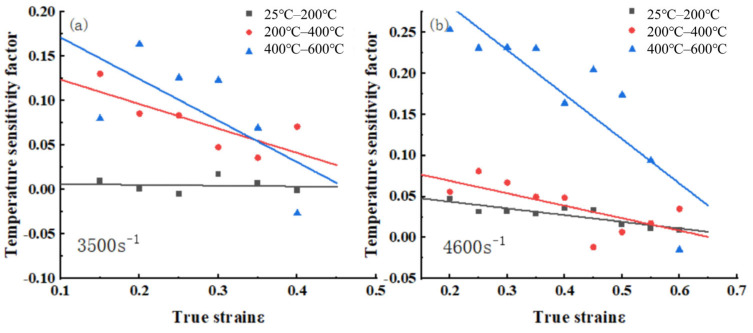
Relationship of temperature sensitivity versus pattern real strain at different temperature change gradients: (**a**) ε = 3500 s^–1^; (**b**) ε = 4600 s^–1^.

To investigate the effect of temperature on flow stress at a constant strain rate, experimental data at strain rates of 3500 s^−1^ and 4600 s^−1^ were used as the basis. The variation in the temperature sensitivity factor with strain is illustrated in Figure 4, showing that $S_{T}$ is influenced by both temperature and strain. As temperature increases, the temperature sensitivity factor generally rises. As strain increases, the temperature sensitivity factor exhibits a noticeable downward trend. This trend is most pronounced between 400 °C and 600 °C. This indicates that as plastic deformation progresses, the dominant softening mechanism of the material changes. In the early stages of deformation, the rheological stress is mainly controlled by thermally activated dislocations, which are highly sensitive to temperature. However, in the high-strain stage, the accumulation of dislocation density and the adiabatic temperature rise together promote the occurrence of strain history-dependent softening mechanisms such as dynamic recovery/recrystallization. These mechanisms weaken the direct dependence of rheological stress on the initial environmental temperature, leading to a decrease in the $S_{T}$ value [18]. Between 25 °C and 200 °C, the temperature sensitivity factor remains relatively stable, with fluctuations but no clear trend. This indicates that strain has less influence on the temperature sensitivity factor at lower ambient temperatures.

### 3.2. Analysis of the Reinforcement Effect of the Strain Rate

#### 3.2.1. Analysis of Stress and Strain Curve of Different Strain Rates at the Same Temperature

**Figure 5:**
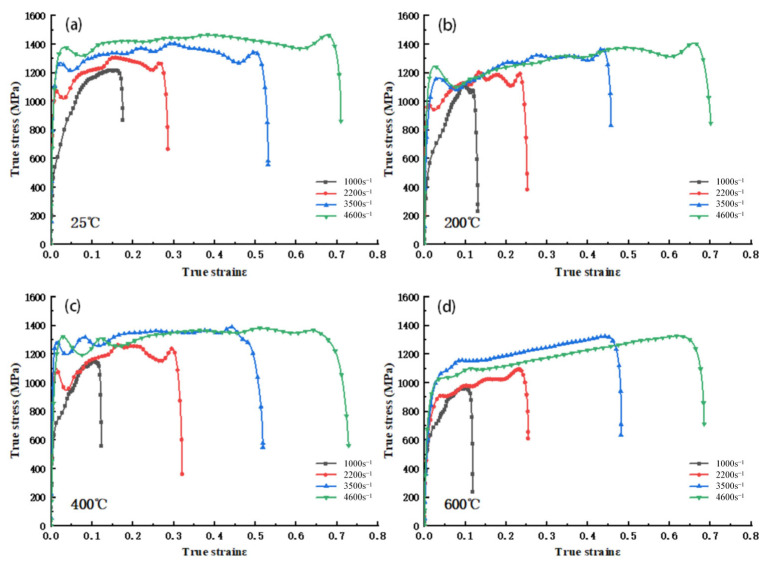
Stress–strain curves of patterns with different strain rates at the same temperature: (**a**) 25 °C; (**b**) 200 °C; (**c**) 400 °C; (**d**) 600 °C.

Figure 5 illustrates the dynamic compressive impact σ−ε curves of 34CrNi3MoA low-alloy steel at various strain rates and ambient temperatures of 25 °C, 200 °C, 400 °C, and 600 °C. It is evident that with increasing strain rates, the flow stress in the yield stage of the low-alloy steel shows a marked upward trend, indicating a significant strain rate strengthening effect. With the escalation of strain rate, the strain-hardening effect of the material exhibits a distinct decreasing trend. This is attributed to the rapid increase in dislocation density in metallic materials at high strain rates, which induces dynamic recrystallization, thereby reducing the strain-hardening effect. Concurrently, higher strain rates result in a more pronounced adiabatic temperature rise. As the adiabatic temperature rise intensifies with escalating strain, the thermal softening effect competes with the strain-hardening effect, thereby causing a reduction in the latter.

#### 3.2.2. Effect of the Strain Rate on the Compressive Strength of the Material

**Figure 6:**
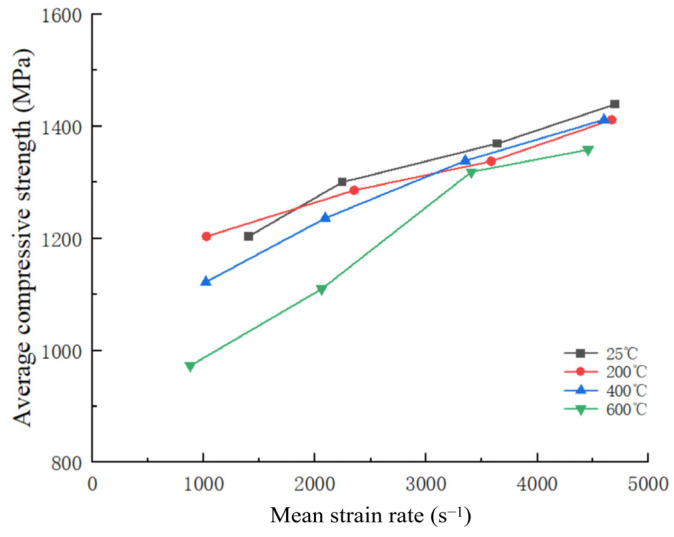
Relation between average compressive strength and average strain rate.

The variation in average compressive strength of 34CrNi3MoA low-alloy steel with respect to strain rate is depicted in Figure 6. At a constant temperature, the compressive strength of 34CrNi3MoA low-alloy steel generally exhibits a significant upward trend with the escalation of strain rate. At an experimental temperature of 600 °C, the compressive strength increases most significantly with the increase in strain rate. The average compressive strength rises from 972.7 MPa (at a strain rate of 1000 s^−1^) to 1358 MPa (at a strain rate of 4600 s^−1^), an increase of 42.39%. At 400 °C, the average compressive strength increases by 25.78%. The increment of residual compressive strength at room temperature and 200 °C is about 20%. This phenomenon can be attributed to the strain rate strengthening effect. At high strain rates, due to dislocation movement, the material’s ability to deform decreases, showing a stronger compressive strength. The residual increment in compressive strength at room temperature and 200 °C conditions did not exceed 20%.

#### 3.2.3. Strain Rate Sensitivity Analysis

To examine the impact of strain rate on the mechanical properties of 34CrNi3MoA low-alloy steel, strain rate sensitivity β is introduced and formulated as per Equation (5).

(5) $$\beta =\frac{\partial \sigma }{\partial ln\dot{\varepsilon }}=\frac{\sigma_{2}-\sigma_{1}}{\ln\left(\frac{{\dot{\varepsilon }}_{2}}{{\dot{\varepsilon }}_{1}}\right)}$$

where ${\dot{\varepsilon }}_{1}$ and ${\dot{\varepsilon }}_{2}$ represent the two strain rates at the same temperature, $\sigma_{1}$ and $\sigma_{2}$ are the true stress values corresponding to strain rates ${\dot{\varepsilon }}_{1}$ and ${\dot{\varepsilon }}_{2}$ at the identical temperature [19,20].

**Figure 7:**
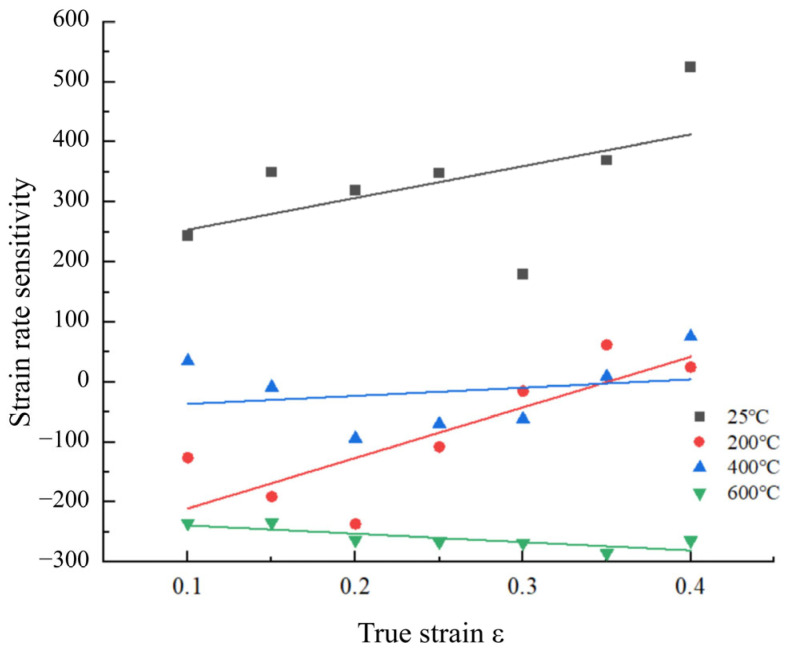
Changes in strain rate sensitivity with pattern strain at different ambient temperatures.

To evaluate the impact of strain rate on the flow stress, experimental data at strain rates of 3500 s^−1^ and 4600 s^−1^ were utilized as the basis. The variation in strain rate sensitivity β with strain is illustrated in Figure 7. At room temperature and 200 °C, the strain rate sensitivity of 34CrNi3MoA low-alloy steel exhibits an increasing trend with increasing true strain. However, when examined individually, there is an initial decrease followed by an increase in strain rate sensitivity. When the experimental temperature is elevated to 400 °C, the strain rate sensitivity shows a similar initial decrease and subsequent increase as observed at 200 °C, but the overall increasing trend is more gradual. At 600 °C, the strain rate sensitivity decreases slowly with increasing strain. This is due to the fact that at the initial stage of plastic deformation, the hardening effect caused by the refinement of the microstructure and the proliferation and entanglement of a large number of dislocations is greater than the thermal softening effect. However, under high strain, the increase in deformation temperature enhances the thermal softening effect, which slows the increase in flow stress. Therefore, there is a phenomenon where materials exhibit strong strain rate sensitivity at low temperatures [21].

### 3.3. Analysis of the Processing-Hardening Effect

**Figure 8:**
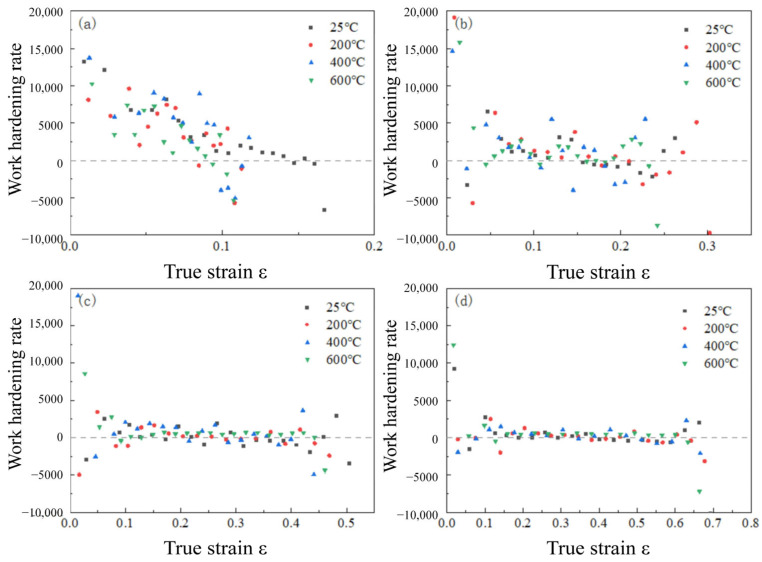
Changes in work-hardening rate with pattern strain at different ambient temperatures: (**a**) ε = 1000 s^–1^; (**b**) ε = 2200 s^–1^; (**c**) ε = 3500 s^–1^; (**d**) ε = 4600 s^–1^.

To investigate the work-hardening effect, the work-hardening rate $Q_{i}$ is defined as the slope of the stress–strain curve for a specific strain change [22,23,24]. Consequently, the hardening rate is calculated as per Equation (6).

(6) $$Q_{i}=\frac{\partial \sigma }{\partial \varepsilon }=\frac{\sigma_{i}-\sigma_{i-1}}{\varepsilon_{i}-\varepsilon_{i-1}},i\geq 1$$

here, $\sigma_{i}$ and $\varepsilon_{i}$ denote the stress and strain at point i, respectively, and the work-hardening rate with respect to strain rate and temperature is illustrated in Figure 8. The work-hardening rate peaks at the onset of plastic deformation and then diminishes rapidly with increasing strain.

The phenomenon of strain hardening is a macroscopic representation of changes that occur in the internal organization of materials at the microscopic level [25]. During deformation, the formation and growth of dislocations, the formation of twins, and the increase in deformation areas within grains that impede dislocation movement enhance the interaction and entanglement between dislocations and surrounding deformation areas. For the low-alloy steel 34CrNi3MoA, the rapid decrease in strain hardening rate with increasing strain rate is due to the extreme activity of dislocation movement under high-strain-rate conditions, where the dislocation entanglement within the material is nearly at its limit even under lower strains, thus suppressing the strain hardening of the material.

## 4. Constitutive Model of 34CrNi3MoA Low-Alloy Steel

### 4.1. Original J-C Constitutive Model for 34CrNi3MoA Low-Alloy Steel

The original Johnson–Cook principal model was employed to fit the dynamic mechanical properties of 34CrNi3MoA low-alloy steel [26,27], as presented in Equation (7).

(7) $$\sigma_{P}=\left(A+B\varepsilon_{P}^{n}\right)\left(1+C\ln\frac{\dot{\varepsilon }}{\dot{\varepsilon_{0}}}\right)\left[1-{\left(\frac{T-T_{r}}{T_{m}-T_{r}}\right)}^{m}\right]$$

where $\sigma_{P}$ denotes the plastic flow stress; $\varepsilon_{P}$ represents the equivalent plastic strain; $\dot{\varepsilon }$ is the equivalent plastic strain rate; $\dot{\varepsilon_{0}}$ is the reference strain rate; $T_{r}$ is the reference temperature; $T_{m}$ refers to the melting point temperature of the material; T is the initial deformation temperature, and A, B, n, C and m are material constants, as presented in Table 3. In the original model, the effects of work hardening, strain rate hardening, and thermal softening are considered separately, neglecting the coupling effects of strain, strain rate, and temperature.

To solve the parameters of the intrinsic model, the common method involves controlling variables and solving them term by term. For 34CrNi3MoA low-alloy steel, room temperature (25 °C) is used as the reference temperature and the reference strain rate is 1400 s^−1^, which is the lowest strain rate observed in the room temperature experiments [28,29]. When temperature and strain rate are set to reference values, the parameters C and m are eliminated, and A represents the true yield strength at these conditions, which is 963 MPa. This allows for the determination of B and n. Given that the plastic strain corresponding to the yield strength is zero, the true yield strengths at different strain rates under the reference temperature can be used to calculate the value of C. Since the first two terms of the Johnson–Cook model remain constant when only the temperature is changed, the ratio of the yield stress at high temperatures to the yield stress at the reference temperature under the same strain rate can be used to determine the value of m. The average value of mmm is obtained by calculating it under multiple strain rate conditions.

**Figure 9:**
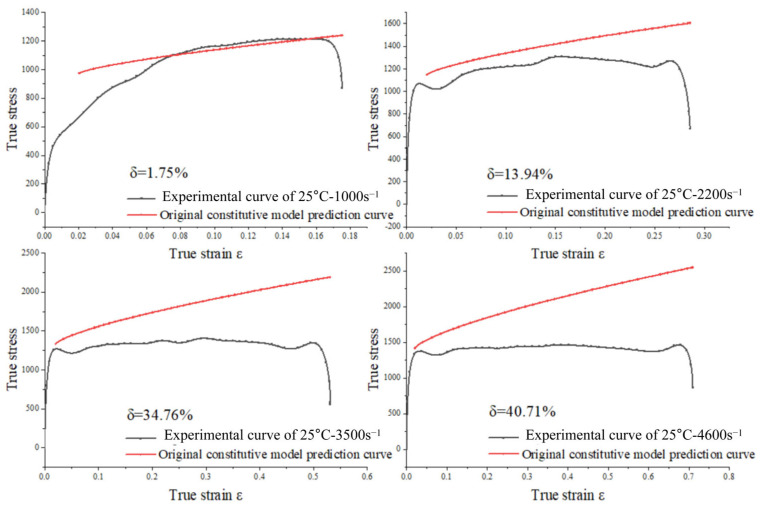
Comparison of the predicted and experimental curves of the original J-C model.

A comparison between the measured results and the predicted results using the original Johnson–Cook model for alloy steel is illustrated in Figure 9. δ represents the magnitude of the error. It is evident that the predictions are accurate only at the reference temperature and reference strain rate. As the disparity between the test strain rate and reference strain rate, and the test temperature and reference temperature increases, the prediction accuracy declines and the prediction error escalates. This indicates that the original Johnson–Cook model cannot adequately characterize the flow behavior of the alloy steel under study. This limitation arises because the original Johnson–Cook model assumes that the effects of work hardening, strain rate hardening, and thermal softening are independent. In reality, the coupled effects of strain rate and temperature on the flow behavior of alloy steel must be considered.

### 4.2. Modification of the Johnson–Cook Constitutive Model

#### 4.2.1. Strain Rate Reinforcement Function of the Coupling Temperature

**Figure 10:**
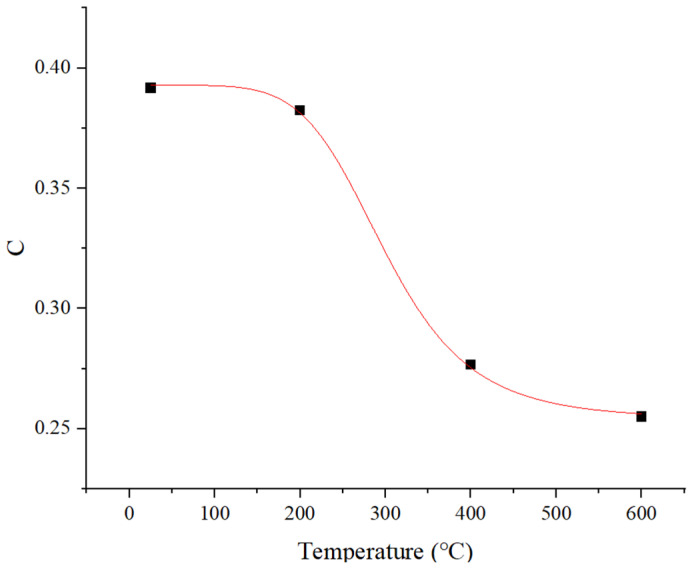
The relationship between the strain rate reinforcement coefficient C and temperature.

Figure 10 illustrates the intricate coupling relationship between the strain rate strengthening effect and temperature, wherein the coefficient associated with strain rate strengthening diminishes as the temperature rises. This observation suggests that the strain rate strengthening effect exhibited by 34CrNi3MoA low-alloy steel progressively attenuates with elevating temperature. The figure further reveals that the variation in the strengthening coefficient with respect to temperature is non-uniform, demonstrating a notable decrease at a specific critical temperature. Owing to the limited experimental data, the precise critical temperature remains indeterminate at this juncture but is estimated to be approximately ($T_{c}$ = 300 °C). Consequently, the function delineated in Equation (8) is employed to describe the aforementioned coupling relationship between temperature and strain rate effects.

(8) $$C\left(T\right)=\frac{c_{1}}{1+{\left(\frac{T}{T_{c}}\right)}^{c_{2}}}+c_{3}$$

where $c_{1}$, $c_{2}$ and $c_{3}$ are fitting parameters derived from experimental data.

#### 4.2.2. Temperature Softening Function Based on the Dynamic Strain Aging Phenomenon

**Figure 11:**
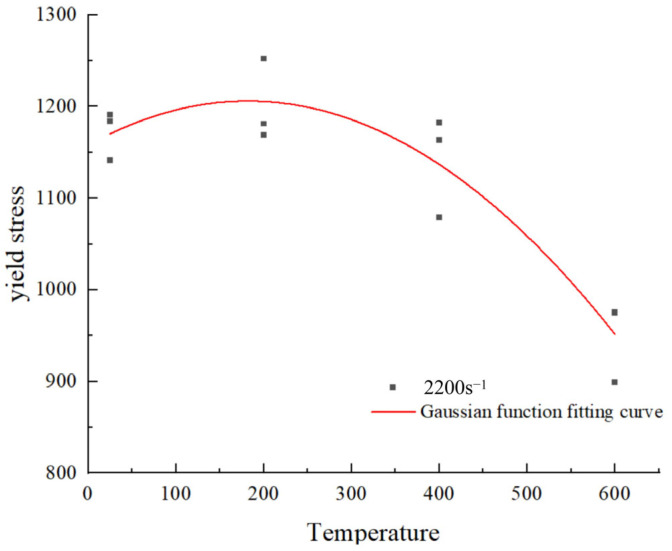
The strain rate of 2200 s^−1^ Time yield stress with temperature.

The trend of yield stress as a function of temperature at a strain rate of 2200 s^−1^ is depicted in Figure 11. A distinct peak in anomalous stress emerges at a strain rate of 2200 s^−1^. This aligns with the type III dynamic strain aging phenomenon observed in the majority of alloys [30]. At this time, soluble atoms such as carbon (C) and chromium (Cr) have sufficient diffusion ability, but cannot fully keep up with the high-speed movement of dislocations, thus forming an unbalanced, periodic solute cluster around the moving dislocations [31]. This cluster exerts a strong ‘pinning’ effect on the dislocations, causing dislocation movement to require greater stress, which macroscopically manifests as an abnormal increase in flow stress with rising temperature, forming a peak on the curve [32]. After the peak, as the temperature continues to rise, atomic diffusion significantly accelerates, the solute cluster becomes loose, and the pinning effect weakens, while thermal softening begins to dominate the deformation process, leading to a rapid decrease in yield stress [33,34]. It is worth noting that the position of this abnormal stress peak is not constant; it has a clear relationship with strain rate and has been observed in the high-strain-rate deformation processes of various other steel materials [35,36].

A modified Johnson–Cook (J-C) constitutive model, as depicted in Equation (9), is proposed based on the principles discussed above [37]. This paper proposes a modified temperature softening term function, $f_{m3}\left(T,\dot{\varepsilon }\right)$, which takes into account the effect of strain rate, as expressed in Equation (10).

(9) $$\begin{array}{c} \sigma =\left(A+B\varepsilon^{n}\right)\left[1+C_{3}\ln{\overset{˙}{\varepsilon }}^{*}+C_{4}\left(\frac{1}{C_{5}-\ln{\overset{˙}{\varepsilon }}^{*}}-\frac{1}{C_{5}}\right)\right]\left\{1-T^{*m}+D\exp\left[-\frac{{\left(T-T_{p}\right)}^{2}}{2d^{2}}\right]\right\} \end{array}$$

(10) $$\begin{array}{c} \left\{\begin{array}{c} T_{p}\left(\dot{\varepsilon }\right)=T_{0}+K{\left(\frac{\dot{\varepsilon }}{\dot{\varepsilon_{0}}}\right)}^{r}\\ f_{m3}\left(T,\dot{\varepsilon }\right)=1-{T^{*}}^{m_{0}}+\mathrm{Dexp}\left[-\frac{{\left(T-T_{p}\left(\dot{\varepsilon }\right)\right)}^{2}}{2d^{2}}\right] \end{array}\right. \end{array}$$

where $T_{p}\left(\dot{\varepsilon }\right)$ represents the peak temperature offset function, characterizing the shift in the position of the anomalous stress peak on the stress-temperature curve as a function of strain rate. Each parameter is experimentally determined. $m_{0}$ represents the temperature softening exponent function, substituting the original temperature softening exponent m in the J-C constitutive model, to dynamically characterize temperature sensitivity across varying strain rates. D and d denote the peak stress-related fitting parameters, determined from empirical data.

#### 4.2.3. Strain Hardening Correction Function for Coupling Strain Rate and Temperature Under Low Strain

Based on the mechanical analysis in Section 3.1.1 and the error analysis of the original J-C constitutive model in Section 4.1, it is evident that the strain-hardening effect is pronounced under reference strain rate conditions. However, the strain-hardening effect weakens under conditions with higher strain rates. There is a discernible trend of the strain-hardening effect diminishing with increasing strain rates. In the original J-C constitutive model, the strain hardening rate remains nearly constant, resulting in an overestimation of the strain-hardening effect at high strain rates. This indicates that the low-alloy steel demonstrates a clear strain hardening–strain rate coupling relationship, necessitating a correction to the strain hardening term in the original J-C constitutive model.

**Figure 12:**
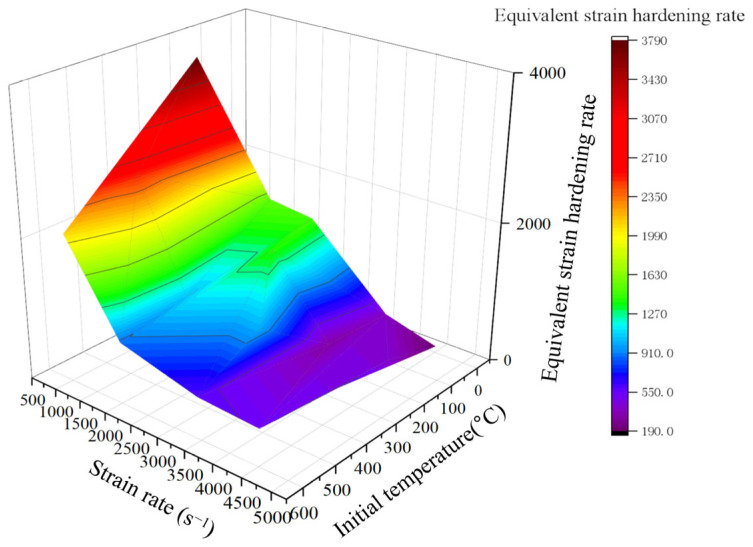
Relations of equivalent strain hardening rate with temperature and strain rate.

The average work hardening rate at low strain values is considered the equivalent strain hardening rate [38]. Figure 12 illustrates the relationship between the equivalent work hardening rate and both the temperature and strain rate, revealing that the strain hardening rate decreases rapidly with increasing strain rate and stabilizes at higher strain rates. Thus, we introduce an equivalent strain hardening rate $Q\left(\dot{\varepsilon }\right)$ expressed in the form of an exponential function. This function is no longer a constant, but a dynamic function that precisely describes the pattern shown in Figure 12. The value of $Q\left(\dot{\varepsilon }\right)$ rapidly decreases from its initial value to a stable value as $\dot{\varepsilon }$ increases, which accurately captures the physical phenomenon shown in Figure 12. This corrected function performs better than the original constitutive model and the common linear correction models.

(11) $$Q\left(\dot{\varepsilon }\right)=y_{0}+{ae}^{\left(\frac{\dot{\varepsilon }-\dot{\varepsilon_{0}}}{b}\right)}$$

where $\dot{\varepsilon_{0}}$ is the reference strain rate of 1400 s^−1^; $y_{0}$, a and b are fitting parameters derived from experimental data.

### 4.3. Modified Constitutive Model Fitting Results and Error Analysis

Based on the coupled temperature-dependent strain rate strengthening function proposed in Section 4.2.1, Section 4.2.2 and Section 4.2.3, the temperature softening function for dynamic strain aging phenomena, and the strain hardening correction function that couples strain rate and temperature at low strains, the original Johnson–Cook (J-C) constitutive model is modified. Equation (12) proposes a modified model incorporating coupled strain, strain rate, and temperature. The relevant parameters of the modified function are detailed in Table 4.

(12) $$\;\;\left\{\begin{array}{c} Q\left(\dot{\varepsilon }\right)=y_{0}+{ae}^{\left(\frac{\dot{\varepsilon }-\dot{\varepsilon_{0}}}{b}\right)}\\ C\left(T\right)=\frac{c_{1}}{1+{\left(\frac{T}{T_{c}}\right)}^{c_{2}}}+c_{3}\\ T_{p}\left(\dot{\varepsilon }\right)=T_{0}+K{\left(\frac{\dot{\varepsilon }}{\dot{\varepsilon_{0}}}\right)}^{r}\\ \sigma_{P}=A\left(1+C\left(T\right)\ln\frac{\dot{\varepsilon }}{\dot{\varepsilon_{0}}}\right)\left[1-{\left(\frac{T-T_{r}}{T_{m}-T_{r}}\right)}^{m}+\mathrm{Dexp}\left(-\frac{{\left(T-T_{p}\left(\dot{\varepsilon }\right)\right)}^{2}}{2d^{2}}\right)\right]+Q\left(\dot{\varepsilon }\right)\varepsilon_{P}^{n} \end{array}\right.$$

**Figure 13:**
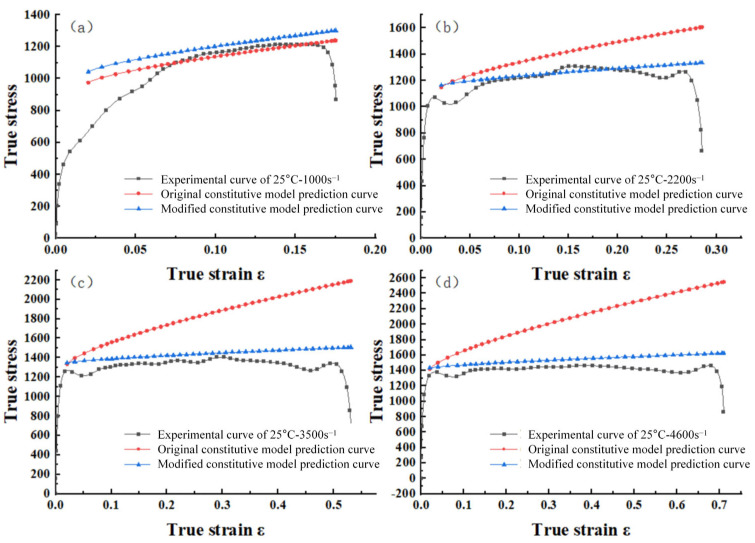
The low-alloy steel stress–strain curves of the J-C model of the original and modified strain rate reinforcement terms at the initial temperature of 25 °C: (**a**) ε = 1000 s^–1^; (**b**) ε = 2200 s^–1^; (**c**) ε = 3500 s^–1^; (**d**) ε = 4600 s^–1^.

The modified Johnson–Cook (J-C) constitutive model was derived based on Equation (12). Four sets of stress–strain curves for the modified J-C constitutive model under consistent temperature and varying strain rate conditions are plotted. These curves are compared with experimental data, as shown in Figure 13. It can be observed that the fit of the modified principal model to the experimental curves exhibits significant improvement over the original principal model presented in Figure 9.

**Figure 14:**
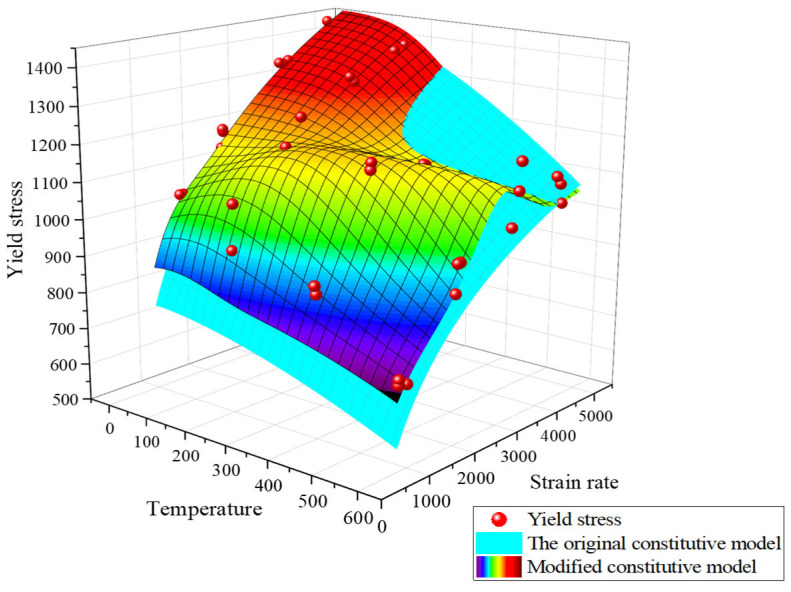
Comparison of the original constitutive model and the yield stress of the experimental data.

Utilizing the parameters mentioned, the three-dimensional surfaces of the yield stresses for both the original and modified constitutive model under varying strain rates and temperatures are plotted. These surfaces are then compared with the actual yield stress data from all experimental results, as depicted in Figure 14. As illustrated in the figure, the modified surface features a noticeable bulge, providing a more accurate depiction of the stress peaks in the low-alloy steel at 200 °C and 2200 s^−1^.

The average relative error $\bar{\delta }$ was employed to quantitatively assess the fitting accuracy of both the original and modified Johnson–Cook (J-C) constitutive models to the material’s stress–strain curves. This error was calculated using Equation (13).

(13) $$\bar{\delta }=\frac{\sum_{i=1}^{N}\left|\frac{E_{i}-Z_{i}}{E_{i}}\right|}{N}\times 100\%$$

where $E_{i}$ represents the experimental actual flow stress, $Z_{i}$ is the model-predicted flow stress, and N denotes the number of data points.

**Figure 15:**
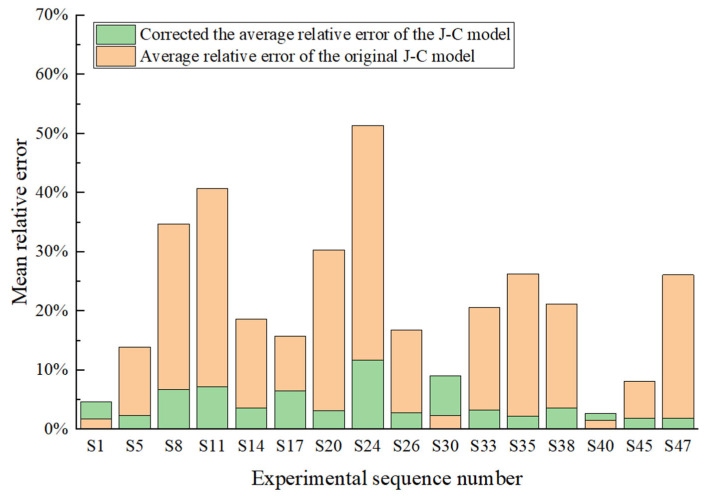
Relative error analysis plot.

The mean relative errors for the original and modified J-C constitutive models across 16 sets of experimental samples are presented in Table 5. As shown in Figure 15, the error range for the original J-C constitutive model spans from 1.49% to 51.39%. The average relative error across different experimental conditions for the original model is approximately 20.63%, indicating a significantly large overall error. This is due to the original J-C constitutive model’s assumption that strain hardening, strain rate strengthening, and thermal softening effects are independent of each other, while it cannot accurately depict the coupling phenomena and nonlinear response of 34CrNi3MoA low-alloy steel under high temperature and high strain rate. The modified J-C constitutive model, which accounts for the coupling relationship between strain rate, strain hardening, and temperature, exhibited an error range of 0.62% to 6.5%, with a mean value of 2.82%. These error results suggest that the modified model proposed in this study provides a more accurate description of the flow stress behavior for 34CrNi3MoA low-alloy steel. Considering the multiple coupling relationships and abnormal stress changes present in these low-alloy steels, this model demonstrates the potential applicability of low-alloy materials with complex mechanical properties in high-temperature and high-strain-rate scenarios such as metal cutting simulation and the dynamic impact response of key components.

## 5. Conclusions

This study investigates the dynamic mechanical response of 34CrNi3MoA low-alloy steel under high-temperature and high-strain-rate conditions, establishing a constitutive model that accurately describes its flow behavior. Based on true stress–true strain data obtained from Separated Hopkinson Pressed Bar (SHPB) experiments, we draw the following key conclusions:

Under low-strain-rate conditions, low-alloy steels exhibit significant strain hardening in the plastic phase as strain increases, followed by an enhanced thermal softening effect. Under high-strain-rate conditions, low-alloy steels display a marked thermal softening effect at low strains, which diminishes as strain increases. At lower temperatures, the rheological stress of low-alloy steel tends to increase significantly with a rising strain rate, while the strain hardening effect tends to decrease significantly with an increasing strain rate.

There is a coupling between the strain rate strengthening term and temperature. The strain hardening effect is very pronounced under low-strain-rate conditions but gradually diminishes as the strain rate increases, indicating a coupling relationship between strain hardening and strain rate.

The modified constitutive model shows a significant improvement in fitting the experimental curves, reducing the error range from the original J-C model’s 1.49% to 51.39% range down to 0.62% to 6.5%. The J-C constitutive model, which takes into account the coupling effects of temperature, strain, and strain rate, effectively describes the flow stress of 34CrNi3MoA low-alloy steel. At the same time, this method has significant engineering implications for studies on similar correction models for other steels.

## Figures and Tables

**Table 1:** Chemical composition of 34CrNi3MoA.

C	Mn	Si	Cr	Ni	P	S	Mo
0.32	0.69	0.17	0.98	2.9	0.005	0.004	0.32

**Table 2:** Design of the SHPB experiment.

Experimental Serial Number	Temperature (°C)	Strain Rate (s^−1^)
S1–3	25	1000
S4–6	25	2200
S7–9	25	3500
S10–12	25	4600
S13–15	200	1000
S16–18	200	2200
S19–21	200	3500
S22–24	200	4600
S25–27	400	1000
S28–30	400	2200
S31–33	400	3500
S34–36	400	4600
S37–39	600	1000
S40–42	600	2200
S43–45	600	3500
S46–48	600	4600

**Table 3:** Original constitutive model parameters.

Parameter Name	A	B	n	C	m	$\dot{\varepsilon_{0}}$	*T*_m_
price	963	1010	0.718	0.39165	1.54	1400	1420

**Table 4:** Table of related parameters of the additional function of strain hardening.

parameter name	A	$c_{1}$	$c_{2}$	$c_{3}$	$T_{c}$	m	D	$T_{0}$	k
price	901	0.14	5	0.26	300	2.08	0.21	−90	249
parameter name	r	d	$y_{0}$	a	b	n	$T_{m}$	$T_{r}$	$\dot{\varepsilon_{0}}$
price	1.01	79.8	279	1375	−1007	0.89	1420	25	1400

**Table 5:** Statistics of prediction relative errors of constitutive models.

Experimental Serial Number	Temperature	Strain Rate	The Original J-C Model $\bar{\delta }$	Correct the J-C Model $\bar{\delta }$
S1	25	1434	1.75%	1.50%
S5	25	2261	13.94%	4.19%
S8	25	3705	34.76%	2.69%
S11	25	4621	40.71%	2.96%
S14	200	1038	18.63%	3.57%
S17	200	2467	15.71%	2.17%
S20	200	3543	30.31%	2.59%
S24	200	4594	51.39%	6.50%
S26	400	1105	16.80%	2.84%
S30	400	2006	2.36%	5.43%
S33	400	3361	20.60%	2.96%
S35	400	4755	26.28%	1.80%
S38	600	990	21.19%	3.24%
S40	600	2014	1.49%	1.22%
S45	600	3438	8.07%	0.62%
S47	600	4515	26.11%	0.76%
mean			20.63%	2.82%

## Data Availability

The original contributions presented in this study are included in the article. Further inquiries can be directed to the corresponding author.
